# Socioeconomic inequalities in health in the context of multimorbidity: A Korean panel study

**DOI:** 10.1371/journal.pone.0173770

**Published:** 2017-03-15

**Authors:** Myung Ki, Yo Han Lee, Yong-Soo Kim, Ji-Yeon Shin, Jiseun Lim, James Nazroo

**Affiliations:** 1 Department of Preventive Medicine, Korea University College of Medicine, Seoul, South Korea; 2 Department of Preventive Medicine, Konyang University College of Medicine, Daejeon, South Korea; 3 Seoul Metropolitan Government Public Health Policy Institute, Seoul, South Korea; 4 Department of Preventive Medicine, School of Medicine, Eulji University, Daejeon, South Korea; 5 Cathie Marsh Institute for Social Research, School of Social Science, University of Manchester, Manchester, United Kingdom; Leibniz Institute for Prvention Research and Epidemiology BIPS, GERMANY

## Abstract

Socioeconomic inequalities in health are commonly known to decrease at late age. Yet, it remains unclear whether socioeconomic inequalities in health at late age appear in relation to multimorbidity, particularly in Korea where social support remains unsatisfactory for older people. Using three waves of Korea Health Panel, data of 19,942 observations with repeated measure were constructed to ensure a temporal sequence between three socioeconomic measures (i.e., poverty, employment status, and education) and multimorbidity with a *t* to *t*+1 year transition. A multilevel multinomial model was applied to quantify the socioeconomic impact across different age, diseases and disease groups, both separately and in combination. There were associations between socioeconomic position (SEP) and multimorbidity, and increasing trends of socioeconomic inequalities not only with greater number of morbidity but also with age. The latter result was only observed with employment status through mid-to-early old age; i.e., between the 40s (odds ratio (OR) = 2.45, 95% confidence interval (CI):1.08–5.57) and 70s (OR = 3.48, 95%CI: 1.24–9.74). The patterns of socioeconomic inequalities in multimorbidity varied for particular pairs of diseases and were stronger in the disease pairs co-occurring with mental and cardiovascular diseases but weaker in the disease pairs co-occurring with cancer. Accumulation of adversity tended to intensify with increase in number of diseases and older age, though this finding was not consistently supported. The labour market should be encouraged to actively participate in actions to promote healthy aging needs to be complemented by the provision of more generous and universal income support to the elderly in Korea.

## Introduction

Multimorbidity, defined as the co-occurrence of two or more chronic diseases, is a common clinical feature, particularly among older people [1, 2]. The substantial burden of multimorbidity on society has drawn due attention. Despite increased recognition of high prevalence of multimorbidity, investigations of socioeconomic inequalities in health have traditionally relied on a single disease approach, with only a handful of studies exploring socioeconomic inequalities in the context of multimorbidity [3–8]. Among such studies, most showed an inverse association between socioeconomic position (SEP) and multimorbidity, regardless of the choice of SEP measures; e.g. education [5, 7, 9], income [3, 8], social deprivation [10, 11] or lifetime SEP measures [9]. However, few studies have disaggregated multimorbidity into multinomial categories with varying numbers of diagnoses [3, 12], instead of using binomial categories (i.e., having vs. not-having multimorbidity), and uncertainty remains regarding whether the strength of socioeconomic association rises with an increase of number of morbidities. Furthermore, previous studies typically included a limited list of chronic diseases(i.e., sometimes six [3] or seven diseases [13]), which has been criticized [14], because a substantial number of people who suffer from less prevalent diseases are not accounted for in analyses [15].

Studies depending on the analysis of the count of chronic diseases were also limited [10], where a score of one is assigned to each disease to provide a total multimorbidity count. With this counting approach, each disease is given the same socioeconomic weight in multimorbidity analysis. In fact, patterns of socioeconomic inequalities in health vary by each single disease both in morbidity [16, 17] and mortality [18, 19]. There is also some evidence that socioeconomic inequalities in multimorbidity are substantially heterogeneous and strong for specific different disease sets. To illustrate, in a recent area-based ecological study, a distinction was made between two major categories of multimorbidity, where the “mental-only” multimorbidity was more strongly associated with SEP than was the “physical-only” multimorbidity [11]. Another study showed large dissimilarities between two different pairs of disease group; one such pair, comprising mental disorder and a pain cluster, was not related to SEP, while another pair, consisting of cardiovascular and metabolic disorders, was related to SEP [7]. Though these studies represent advances in quality in multimorbidity research, analyses conducted therein were limited to a few pre-selected combinations of diseases, with other disease combinations being largely neglected. Thus, it still lacks details about which specific pairs of disease group are more influenced by SEP than others.

The existence of socioeconomic inequalities in health at old age is well established [20–22]. However, there has been a debate on whether socioeconomic inequalities in health increase or decrease with age. To summarise, some studies argue that socioeconomic inequalities in health persist as advantages and disadvantages accumulate over the lifespan [23], while others support that socioeconomic inequalities in health become gradually smaller, mainly due to two levelers; i) biological declining is fairly even among the elderly population and ii) attenuation of the socioeconomic gap due to social security, which is somewhat favourable to the old generation in most developed countries. In Korea, the latter explanation does not apply, because public spending for the elderly is quite low compared to the average of Organization for Economic Cooperation and Development (OECD) countries [24], while the rate of old age poverty is highest among those countries [25]. Notwithstanding this, in Korea, the proportion of the elderly population who are engaged in economic activity was highest among OECD countries [26], often in the form of non-standardized jobs, bridge jobs, and part-time roles. This suggests that Korea has different SEP trajectories at old age and may be a suitable place to explore two competing explanations for old age socioeconomic inequalities in health.

There is general agreement that relying on a distal SEP measure, such as educational attainment, can be misleading, since the ability of the measure in sensing socioeconomic inequalities in health among the elderly people may be diluted with a lengthy time gap after completion of education. Thus, we used three SEP indicators, measured at proximal (relative poverty and employment status) and distal (educational attainment) time points, to ensure that we capture the actual impact of SEP.

Given the currently insufficient understanding of the association between SEP and multimorbidity, we explored whether 1) socioeconomic inequalities in health increase with an increase in the number of diseases; 2) socioeconomic inequalities are steepened when based on a particular pair of diseases; and 3) socioeconomic inequalities in health are larger among older people than younger people.

## Methods

### Study population

The Korea Health Panel (KHP) is an ongoing longitudinal survey of a nationally representative Korean population. For the present study, the participants were derived from three consecutive years’ KHP data between 2009 and 2011 (i.e., from the second to fourth waves), where the definition of multimorbidity was consistent. About 18,000 individuals were re-interviewed during this period. Initially, 33,601 observations with age 30 and over, with no missing values on any of variables in the analysis were included from the three waves. Data were reconstructed to ensure temporal order between independent variables in year *t* and dependent variables in year *t*+1(two observations per each individual). Finally, 19,942 observations from 9,971 individuals were included in the analysis. The study was exempted from ethical approval of the Institutional Research Board at Eulji University, as the current study is a secondary data analysis of an anonymous sample with no personal identifier.

### Measures

Participants were asked to answer an open-ended question to list all diseases for which they received a diagnosis from a physician. This resulted in a comprehensive list of about 370 diseases. The list was reviewed to select chronic diseases in accordance with the Chronic Condition Classification (CCC) [27, 28], which is widely used to assess the chronicity of a condition. Dementia, bipolar disorder, anxiety disorder, and sleep disorder, which are not covered by the CCC, were additionally included, resulting in a total of 66 chronic diseases for the consideration in this study (S1 Table).

SEP measures included educational attainment, employment status and relative poverty. Relative poverty was defined as having an income of less than half of the median equivalized household income, which was calculated as the annual household income divided by the square root of the number of household members. Employment status was assessed as two categories; employed vs. non-employed. The employed group consisted of those who were employed or self-employed, including unpaid family workers. The non-employed group comprised those who were retired, unemployed, a student, on long-term sick leave, involved in house work or in family care. Educational attainment was categorized into two groups but differently by age groups, because of large differences in the distribution of educational attainment according to generations. For those aged less 60 years, only university education was coded as a higher level, while for those aged 60 and more, participants who were educated higher than middle school education were coded as a higher level of education. Primarily for employment status but also for general comparisons between measures, we did not extend the analysis to participants over the age of 85 years, where more than 90 percent were no longer part of the labour force.

A range of relevant socio-demographic and behavioural covariates were identified from the available literature [1, 29]. Marital status was assessed as being with vs. being without spouse (separated, divorced, or widowed). Four variables related to the health behaviour were included: smoking (non- or ex-smokers vs. current smokers), drinking (≤1 occasion vs. ≥2 occasions per week), moderate or vigorous physical activity (< 1 occasion vs.≥ ococcasion per week), and obesity status (normal (body mass index (BMI) < 25kg/m^2^) vs. overweight or obese (BMI ≥ 25kg/m^2^)).

### Statistical analysis

Associations between SEP measures and multimorbidity were assessed using a multinomial multilevel model (random intercept model) to consider the structure of panel data with repeated measurements. Differences in health equalities over age groups were assessed in a descriptive way by comparing odds ratios (ORs) obtained from separate analysis of six age groups (i.e., 30s, 40s, 50s, 60s, 70s and 80s). We examined differences in the associations between socioeconomic factors and subtypes of multimorbidity, defined as pairs of co-occurring diseases and disease groups. For this analysis, 66 diseases were classified into seven diseases and disease groups to increase the number of observations in each group. Diseases and disease groups were chosen with the consideration of clinical significance, frequency of disease and statistical applicability. Some diseases were combined into a single category when they were rare, despite the merit in preserving each disease separately and to test them simultaneously. When a disease fell simultaneously into more than one disease (groups), it was assigned to a category according to the following order; cancer, mental disease, respiratory disease, cardiovascular disease (CVD), diabetes, hypertension and other diseases. As a result, a new multinomial dependent variable was derived with eight categories; no index disease, a single disease (index disease (group)), and pairs between the index disease (group) and six diseases and disease groups (polytomous approach). Further details are available in S2 Table. Multinomial multilevel analysis, to analyse repeated measurements (*t*: level 1) nested within individuals (*i*: level 2), was then separately modelled for each index disease (resulting in 18 models distinguished by six index disease (groups) and three SEP measures), while controlling for age and sex. Age was included as a continuous covariate. To elucidate the structure of this model, a two-level model with *k* categories of multinomial responses (s = 1, …, k) at time *t* can be expressed by an equation [30]:

$$Log\left(\frac{P_{t+1i}^{k}}{P_{t+1i}^{r}}\right)=\beta_{0i}^{\left(k\right)}+\beta_{1i}^{\left(k\right)}{SEP}_{ti}+\beta_{2i}^{\left(k\right)}{Cov}_{ti}+u_{i}^{\left(k\right)}$$

where the expected probability for a category *k* (*P*_*ti*_^(*k*)^) of individual *i* was quantified as a function of SEP, between year *t* and year *t*+1. A series of *k*-1 equations was formatted compared to the reference category (*s* = *r*). Each response (*k* superscript) was denoted with subject-specific intercepts and coefficients and a subject-specific random effect (*u*_*i*_^(*k*)^) was allowed to account for variation within an individual. SEP and Cov represent socioeconomic position and covariates for adjustment. When data convergence was not achieved because of a small number of observation for some categories, the smallest category of the dependent variable was omitted to reduce the number of random parameters. Socioeconomic differences associated with each disease were further assessed by comparing estimates obtained in a separate analysis of multilevel logistic model with binomial outcomes (i.e., having vs not-having an index disease (group); dichotomous approach). SAS software (ver. 9.3; SAS Institute, Inc., Cary, NC, USA) was used for data construction and bivariate analysis and MLwiN software (ver. 2.12; Centre for Multi-level Modelling, Bristol, UK) was used for the analysis of multilevel model. Because quasi-likelihood procedures for a multinomial outcome are unreliable in MLwiN, the Markov Chain Monte Carlo method was applied to estimate model parameters after obtaining starting values from corresponding quasi-likelihood procedures [31]. A burn-in of 3,000 and chain length of 30,000 runs were used to ensure the stability of the results.

## Results

Multimorbidity was much more prevalent in the elderly, compared to the middle-aged group (Table 1). While only 5.4% of the middle-aged group had three or more chronic diseases, the prevalence reached 37.3% in the elderly group. On average, the elderly individuals had two or more chronic diseases. The elderly individuals were typically of lower SEP than the middle-aged individuals. 37.1% of the elderly individuals versus 8% of the middle-aged individuals were poor; likewise, 53% and 28% were non-employed, and 74% and 64% were less educated, respectively.

**Table 1:** Socio-demographic and health behavioural characteristics of study participants from the Korea Health Panel members (2009–2011) by age groups.

	Middle-aged (30–59)		Elderly (60-)			Total
	N	%	N	%	p-value*	% or Mean(SD)
Gender						
Male	6,175	47.3	3,067	44.6		46.3
Female	6,885	52.7	3,815	55.4	<0.001	53.7
Age, Mean (SD)	13,060	44.7(8.0)	6,882	69.4(6.5)		53.2(13.9)
Number of diseases						
No	8,398	64.3	1,120	16.3		47.7
1	2,839	21.7	1,606	23.3		22.3
2	1,119	8.6	1,591	23.1		13.6
≥3	704	5.4	2,565	37.3	<0.001	16.4
Number of diseases over two years, Mean (SD)						
2010	6,614	0.5(0.9)	3,357	2.1(1.6)		1.0(1.4)
2011	6,446	0.6(1.0)	3,525	2.3(1.7)		1.2(1.5)
Educational attainment						
High	4,714	36.1	1,752	25.5		32.4
Low	8,346	63.9	5,130	74.5	<0.001	67.6
Poverty						
Non-poor	11,988	91.8	4,327	62.9		81.8
Poor	1,072	8.2	2,555	37.1	<0.001	18.2
Employment status						
Employed	9402	72.0	3140	46.4		62.9
Non-employed	3658	28.0	3622	53.6	<0.001	37.1
Marriage						
With spouse	11,268	86.3	5,036	73.2		81.8
No spouse	1,792	13.7	1,846	26.8	<0.001	18.2
Smoking						
Non- or ex-smokers	9,608	73.6	5,653	82.1		76.5
Current smokers	3,452	26.4	1,229	17.9	<0.001	23.5
Alcohol drinking						
≤ 1 occasion per week	9,837	75.3	5,606	81.5		77.4
≥ 2 occasions per week	3,223	24.7	1,276	18.5	<0.001	22.6
Moderate or vigorous physical activity						
< 1 occasion per week	6,548	50.1	2,395	34.8		44.9
≥ 1 occasion per week	6,512	49.9	4,487	65.2	<0.001	55.2
Obesity status (BMI)						
Normal(<25kg/m^2^)	9707	74.3	5134	74.6		74.4
Overweight or Obese(≥25kg/m^2^)	3353	25.7	1748	25.4	0.67	25.6

Note 1:Row frequency (%), apart from age and number of diseases (Mean (SD, standard deviation)). Note 2: For multimorbidity measures, data were pooled over a period between 2010 and 2011, but for all other measures between 2009 and 2010.

*p-value was obtained from Chi-square test.

The distribution of the number of diseases according to subject characteristics is shown in Table 2. Proportions of individuals having multimorbidity increased with age, peaked in the 70s then decreased (for ≥ 3 diseases; 46% in the 70s vs. 42.9% in the 80s). Those with a lower SEP (i.e., lower income, non-employed, and lower education) tended to have more diseases. For example, among those with a lower income, 33% had three or more diseases, compared to 12.6% of the non-poor individuals.

**Table 2:** Bivariate association between socio-demographic factors(t year) and number of chronic disease (t+1 year)in the Korea Health Panel members (2009–2011).

	Total	Number of diseases				p-value*
		0	1	2	3+	
Gender						
Male	9242	53.6	21.9	12.5	12.0	
Female	10700	42.7	22.6	14.5	20.2	<0.001
Age groups						
30s	4043	80.6	16.0	2.7	0.7	
40s	4943	69.9	21.2	6.4	2.5	
50s	4074	41.4	28.0	17.0	13.6	
60s	3800	19.5	26.0	23.8	30.6	
70s	2569	12.5	19.0	22.5	46.0	
80s	513	10.9	25.2	21.1	42.9	<0.001
Educational attainment						
High	6466	59.8	20.7	9.5	10.0	
Low	13476	41.9	23.0	15.6	19.5	<0.001
Poverty						
Poor	3627	52.7	22.5	12.2	12.6	
Non-poor	16315	25.5	21.2	20.0	33.4	<0.001
Employment status						
Employed	12542	55.6	22.3	11.9	10.2	
Non-employed	7280	34.7	22.1	16.3	26.9	<0.001

*p-value was obtained from Chi-square test.

Associations between each SEP measure and number of chronic diseases (i.e., 0 to ≥ 3) varied across SEP measures and age groups (Table 3). Stronger associations were generally observed with more diseases, particularly in 50s, 60s, and 70s, but the strongest associations were observed at 30s for poverty (OR = 5.78, 95% confidence interval (CI):1.16–28.9) and employment status (OR = 4.21, 95% CI: 1.12–15.9). The association of employment status with disease status partially showed a progressively increasing trend up to 70s both in single morbidity (OR = 2.08, 95% CI: 1.06–4.08) and multimorbidity (OR = 3.48, 95% CI: 1.24–9.74), but then a decreasing trend was seen in their 80s. This trend was not observed for poverty or education.

**Table 3:** Odds ratio (95% confidence interval)*of socioeconomic factors (t year) for predicting number of chronic multimorbidity (t+1 year) from multinomial multilevel models fitted to Korea Health Panel (2009–2011), stratified by each socioeconomic measures and age groups.

	Number of diseases			
	0	1	2	3+
Poverty				
30s	Ref	1.10(0.53, 2.30)	2.06(0.48, 8.74)	**5.78(1.16, 28.9)**
40s	-	1.19(0.71, 1.97)	1.71(0.76, 3.83)	**3.77(1.06, 13.4)**
50s	-	0.97(0.57, 1.65)	1.52(0.80, 2.89)	2.20(0.96, 5.02)
60s	-	1.37(0.83, 2.28)	**1.85(1.08, 3.17)**	**2.31(1.28, 4.18)**
70s	-	0.97(0.53, 1.78)	1.15(0.64, 2.06)	1.06(0.52, 2.15)
80s	-	2.18(0.18, 27.0)	1.48(0.17, 12.7)	1.66(0.36, 7.69)
Employment status				
30s	Ref	1.25(0.89, 1.74)	1.03(0.40, 2.69)	**4.21(1.12, 15.9)**
40s	-	1.34(0.94, 1.90)	**1.82(1.16, 2.86)**	**2.45(1.08, 5.57)**
50s	-	**1.48(1.03, 2.14)**	**1.75(1.18, 2.59)**	**3.26(1.61, 6.61)**
60s	**-**	**1.73(1.12, 2.67)**	**2.37(1.31, 4.27)**	**2.96(1.38, 6.37)**
70s	-	**2.08(1.06, 4.08)**	**2.80(1.27, 6.17)**	**3.48(1.24, 9.74)**
80s	-	2.14(0.67, 6.86)	2.48(0.70, 8.82)	**2.86(1.02, 8.05)**
Educational attainment				
30s	Ref	0.85(0.56, 1.27)	1.82(0.70, 4.75)	1.70(0.77, 3.76)
40s	-	1.29(0.87, 1.90)	**1.71(1.03,2.84)**	**2.19(1.19, 4.05)**
50s	-	1.20(0.75, 1.92)	**2.32(1.25, 4.28)**	2.01(0.92, 4.41)
60s	-	**1.71(1.02, 2.87)**	**1.90(1.09, 3.32)**	**2.23(1.10, 4.55)**
70s	-	1.10(0.50, 2.41)	0.96(0.66, 1.39)	2.47(0.56, 10.9)
80s	-	1.83(0.14, 23.5)	1.65(0.10, 25.9)	2.13(0.65, 6.99)

Note 1: All models were adjusted for gender, marital status, smoking, drinking, physical activity and BMI. Note 2: Significant odds ratios are in bold.

*Associations (Odds Ratios (95% Confidence Interval)) were estimated using multinomial multilevel model, separately for each socioeconomic measure and age groups (total 18 models).

Differences in the degree of socioeconomic influence on multimorbidity were separately assessed; first, disease status was identified as having vs. not having any disease (dichotomous categorization) and, second, disease status was unfolded into no disease, single and pairs of diseases (polytomous categorization) with adjustment for age and sex (Table 4). When a disease status was approached in a dichotomous manner, associations between mental disease and all three SEP measures were observed. However, respiratory and cardiovascular diseases were positively associated with two SEP measures (poverty and employment status). Cancer and hypertension were associated with one SEP measure (employment status and education, respectively). When a disease status was disaggregated into single disease and disease pairs, socioeconomic gradients were typically larger for the disease pairs that co-occurring with mental and cardiovascular diseases but smaller for the pairs that co-occurring with cancer. For example, CVD-mental disease pair has associations with poverty (OR = 2.59, 95% CI: 1.03–6.48) and employment status (OR = 4.45, 95% CI: 1.13–17.5), and CVD-respiratory disease pair was also associated with poverty (OR = 2.66, 95% CI: 1.10–6.44) and employment status (OR = 2.69, 95% CI: 1.08–6.69), but no associations were seen for any disease pair that co-occurring with cancer. Among the SEP measures, significant associations were most commonly observed for employment status measures.

**Table 4:** Odds ratio (95% confidence interval)*of socioeconomic factors for each disease groups and pairs between six disease groups from multinomial multilevel models fitted to Korea Health Panel (2009–2011).

	Cancer	Mental disease	Respiratory disease	Cardiovascular diseases	Diabetes	Hypertension
*Poverty*						
Dichotomous categorization^†^	1.66(0.57,4.83)	**2.69(1.18, 6.12)**	**1.39(1.01, 1.91)**	**1.45(1.04, 2.01)**	1.27(0.97, 1.67)	1.26(0.85, 1.87)
Polytomous categorization^†^						
Index disease group only	1.26(0.67, 2.37)	**2.46(1.05, 5.76)**	**1.31(1.02, 1.67)**	1.34(0.82, 2.19)	0.82(0.57, 1.18)	1.28(0.67, 2.45)
Cancer	-	2.93(0.49, 17.4)	1.53(0.24, 9.87)	2.39(0.44, 12.9)	0.97(0.42, 2.27)	1.13(0.53, 2.38)
Mental disease	2.74(0.15, 49.11)	-	2.25(0.43, 11.7)	**2.59(1.03, 6.48)**	2.01(0.93, 4.35)	**2.41(1.07, 5.43)**
Respiratory disease	1.72(0.11, 27.0)	3.72(0.32, 42.6)	-	**2.66(1.10, 6.44)**	2.09(0.47, 9.21)	1.39(0.72, 2.70)
Cardiovascular diseases	2.06(0.42,10.0)	3.13(0.91, 10.8)	**1.82(1.03, 3.21)**	-	1.15(0.69, 1.93)	1.02(0.56, 1.86)
Diabetes	0.87(0.33, 2.32)	2.01(0.91, 4.43)	2.02(0.40, 10.15)	1.12(0.66, 1.91)	-	1.14(0.82, 1.60)
Hypertension	1.04(0.36, 3.06)	**2.14(1.08, 4.27)**	1.40(0.93, 2.11)	1.33(0.88, 2.01)	**1.40(1.02, 1.93)**	-
Others	1.96(0.85, 4.53)	**3.03(1.17, 7.84)**	1.30(0.88, 1.92)	**1.77(1.03, 3.03)**	1.32(0.80, 2.16)	1.35(0.91, 1.99)
*Employment status*						
Dichotomous categorization^†^	**2.22(1.04, 4.72)**	**3.08(1.19, 7.98)**	**1.46(1.04, 2.05)**	**2.31(1.10, 4.88)**	**1.28(1.11, 1.46)**	1.10(0.93, 1.29)
Polytomous categorization^†^						
Index disease group only	1.67(0.93, 3.00)	**2.69(1.20, 6.06)**	**1.38(1.10, 1.73)**	**1.86(1.03, 3.37)**	0.74(0.43, 1.27)	1.21(0.71, 2.07)
Cancer	-	2.75(0.31, 24.8)	1.65(0.49, 5.51)	2.59(0.74, 9.02)	2.46(0.97, 6.23)	1.48(0.49, 4.45)
Mental disease	4.08(0.55, 30.6)	-	3.42(0.56, 21.1)	**4.45(1.13, 17.5)**	**2.36(1.08, 5.14)**	1.89(0.98, 3.64)
Respiratory disease	2.03(0.43, 9.72)	NA^‡^	-	**2.69(1.08, 6.69)**	1.83(0.53, 6.26)	1.49(0.85, 2.61)
Cardiovascular diseases	2.49(0.53, 11.6)	**5.43(1.31, 22.5)**	1.87(0.95, 3.65)	-	**1.69(1.18, 2.41)**	**1.93(1.08, 3.46)**
Diabetes	**3.37(1.05, 10.8)**	3.54(0.87, 14.4)	1.68(0.55, 5.15)	**2.38(1.06, 5.36)**	-	1.23(0.88, 1.72)
Hypertension	1.26(0.67, 2.39)	1.57(0.93, 2.66)	1.42(0.95, 2.12)	**2.14(1.14, 4.01)**	1.25(0.94, 1.67)	-
Others	**1.93(1.06, 3.53)**	**3.28(1.12, 9.62)**	1.41(0.86, 2.31)	2.23(0.96, 5.20)	**1.29(1.02, 1.64)**	0.89(0.72, 1.10)
*Education*						
Dichotomous categorization^†^	1.33(0.61, 2.94)	**1.42(1.02, 1.97)**	1.39(0.87, 2.23)	1.35(0.88, 2.09)	**1.26(1.01, 1.58)**	**1.17(1.00, 1.36)**
Polytomous categorization^†^						
Index disease group only	1.42(0.59, 3.44)	1.23(0.96, 1.58)	1.09(0.81, 1.45)	1.21(0.79, 1.86)	0.96(0.87, 1.04)	1.13(0.63, 2.03)
Cancer	-	1.73(0.30, 10.1)	1.57(0.36, 6.88)	1.19(0.34, 4.21)	1.12(0.60, 2.09)	0.83(0.44, 1.56)
Mental disease	2.09(0.34, 12.9)	-	NA^‡^	1.49(0.94, 2.37)	1.23(0.84, 1.82)	**1.73(1.16, 2.56)**
Respiratory disease	1.69(0.39, 7.40)	2.23(0.35,14.3)	-	2.05(0.84, 5.03)	2.33(0.62, 8.77)	1.59(0.93, 2.69)
Cardiovascular diseases	1.30(0.38, 4.43)	**1.71(1.04, 2.79)**	1.49(0.80, 2.77)	-	**1.41(1.06, 1.88)**	**1.28(1.01, 1.62)**
Diabetes	1.31(0.69, 2.51)	1.14(0.78, 1.66)	2.03(0.48, 8.65)	**1.35(1.02, 1.80)**	-	1.15(0.89, 1.49)
Hypertension	0.57(0.27, 1.21)	**1.39(1.13, 1.70)**	1.33(0.95, 1.85)	**1.37(1.00, 1.88)**	**1.49(1.04, 2.14)**	-
Others	1.33(0.94, 1.88)	1.45(1.00, 2.10)	1.13(0.61, 2.08)	1.49(0.86, 2.60)	1.15(0.83, 1.59)	1.25(0.98, 1.58)

Note 1: All models were adjusted for age and gender. Note 2: The same reference category (i.e. free of each index disease group) was applied to both dichotomous and polytomous approaches. Note 3: When a disease fell into more than one category, it was assigned to a category according to the following order; cancer, mental disease, respiratory disease, cardiovascular disease, diabetes, hypertension and other diseases. Note 4: Significant odds ratio are in bold.

*Associations (Odds Ratios (95% Confidence Interval)) were estimated using multinomial multilevel model, separately for each socioeconomic measures and index disease groups by two approaches (i.e. dichotomous and polytomous approaches)(total 36 models).

^†^Disease status was assessed as dichotomous (i.e. have disease vs no disease) or polytomous categories (i.e. no disease, index disease group, and pairs of diseases).

^‡^NA: the category was omitted when model convergence was not satisfied to simplify the model.

## Discussion

### Main findings

In this large population-based longitudinal study, socioeconomic inequalities were observed for multimorbidity; i.e., a lower SEP was predictive of worse multimorbidity status. Socioeconomic inequalities increased with a greater number of diagnoses in all age groups. Multimorbidity prevalence was higher among the old-aged than mid- to late-adulthood groups and socioeconomic inequalities were generally larger among the 70s but then was smaller in the 80s. Socioeconomic inequalities were greater for the disease pairs when co-occurring with mental and cardiovascular diseases, but less with cancer. The choice of SEP measures and the type of disease matters to the associations and the stronger socioeconomic differences were observed when employment status measure was used.

### Methodological considerations

One strength of this study was the expansion of the traditional approach to encompass multimorbidity; quantitatively as a continuous status (ranging from 0 to ≥3) rather than bivariate cut-off and qualitatively as heterogeneous disease sets rather than a single multimorbidity index (i.e., count). Second, owing to the further differentiation of multimorbidity measures (though it was limited to disease pairs), we were partly able to widen the scope of health inequalities research area. This was related to the potential of multilevel multinomial modelling, which makes it possible to preserve and test each pair of diseases separately and to present the heterogeneous characteristic of socioeconomic inequalities in multimorbidity. Third, we were able to assess socioeconomic inequalities in multimorbidity via multiple ways by taking advantage of three SEP measures. Regarding the use of SEP measures in older populations, it has been argued that a single SEP measure is unlikely to capture the multidimensional nature of socioeconomic relations [32].

We also acknowledge some limitations. First, some disease categories (e.g. mental disease) comprised small numbers of patients and produced estimates with large variances, though data pooling over three years offered a better opportunity for dealing with rare diseases. In addition, the combined categories inevitably neglected the considerable heterogeneity within the domain and yielded overall effects (Table 4). This suggests the need for future research with better differentiation of individual disease. Second, though the current study was based on longitudinal structure with one year interval, we did not rule out cases that occurred before the time-frame of this study, while no consideration was taken for onset and duration of disease. Nevertheless, compared to cross-sectional data, panel data with repeated measures allows gains in estimation by partly adjusting for pre-existing relations with controlling for unobserved heterogeneity [33]. The third limitation is related to the inability of this study to observe whether health inequalities widen or narrow in late age, due to the short follow-up period. The current approach was partly able to address the issue by comparing health inequalities across different age groups within a population, instead of examining long-term changes of health inequalities within a cohort.

### Interpretation and comparison with previous studies

Socioeconomic gradients in multimorbidity were observed previously [3–8]. This study further confirms that the gradients are steeper with an increased number of diseases, which was seen across age groups. The progressive increase in socioeconomic inequalities with increasing multimorbidity suggests that the associations between socioeconomic condition and multimorbidity is not simple, but rather reflect a series of cumulative process of advantages and disadvantages; e.g. the adversity associated with a lower SEP negatively affects the course of disease development such that health inequalities widen with an increasing number of diagnoses [34]. This is in agreement with prior studies, in which those with a lower SEP showed inferior long-term management results such as complications, disease severity, associated disabilities, and terminal care. A potential explanation for this finding is that a lower SEP tends to exert negative impacts on various points in the course of long-term management of chronic diseases and leads to delays in seeking treatment, underutilization of care services, and reduced participation in regular follow-up care [35].

Traditionally, socioeconomic inequalities in health have been approached on the assumption that the disease under investigation is a single homogeneous entity but the current study shows that there was substantial heterogeneity when diseases were unfolded into several sets of multimorbidity. It was previously noted that socioeconomic inequalities vary by individual diseases. For example, larger socioeconomic gradients were observed for stroke, diabetes and arthritis, while no gradients were observed for cancer and kidney diseases and reversed gradients were observed for allergy [36]. The current study extended this perspective by exploring underlying details of multimorbidity and found that socioeconomic impact was greater on some sets of disease pairs; i.e., larger socioeconomic gradients for the disease pairs that included mental and cardiovascular disease but smaller gradients for the disease pairs that included cancer. The smaller gradients for the disease pairs that included cancer may be due to differences in the degree of socioeconomic burden across types of cancer; for example, breast and prostate cancer have been reported to show a weak association with SEP [37, 38]. This finding has important policy implications in relation to the critical illness insurance policy launched by Korean government in 2013. This scheme provides special benefits to patients with one of the four major target diseases (i.e., cancer, cerebrovascular, cardiac and rare and incurable diseases) and helps relieve the financial strain of the patients, particularly those at the lower SEP level. Though the current study did not address the issue, this scheme may require due consideration on types of cancer to address fair sharing of benefits due to critical diseases.

Increases in socioeconomic gradients up to the 70s were observed in our study, but only for employment status, and not for poverty or educational attainment. Similar findings have been reported elsewhere [39, 40], though other studies reported declining health inequalities in later life. Some of these disparities may be attributed to differences in study design; studies using a simple age grouping (e.g., young vs. old), focusing on mortality outcomes, using a cross-sectional design without accounting for longer term trajectories, or employing more distal SEP measures such as educational attainment are more likely to observe declining health inequalities with older age [41, 42]. In addition, studies conducted in countries such as the US, where social benefits are more readily available to older individuals, tend to have lower health inequalities at old age [43].

As such, the findings of the present study should be viewed in a Korean context. First, the strong association between employment status and multimorbidity at old age maybe due to a worsening of socioeconomic condition in this group, since old age increase the risk of income poverty among the Korean elderly, despite their highest employment rate among OECD countries. Thus, working into old instead of retiring may deliver health benefits by buffering against the effects of lower income as well as providing the inherent health benefits associated with employment. Second, an additional widening of the socioeconomic gap between the employment and the non-employment may arise from health selective mobility: i.e. health may exert influence on subsequent changes in SEP. This mobility is known to primarily occur during the entry into and exit from employment (health selection) [44]. If this is a main reason, the health gaps indicate that the flexibility in accommodating to and staying in the employment for those with health problems is insufficient. Further researches are required to estimate the specific contribution of these two explanations.

Interestingly, results showed the weakest association between SEP and health in the oldest ages (≥80 years) as shown in prior studies including a review [45]. In this regards, part of this smaller health inequalities may be attributable to selective survival; i.e. the sicker from the lower SEP are more likely to select out of population. Moreover, the biological decline which occurs commonly in later life may offset health variation across socioeconomic groups. In contrast, the magnitude of health inequalities was strongest in the youngest age group (30s), consistent with previous literature [46, 47]. It may be because having chronic diseases at age 30s is infrequent, but once they occur, the health impact is more intensified along the lowest end of socioeconomic distribution. This finding also suggests that economic participation is pretty much dependent on health status particularly in early age than in later age with better buffering through family and social support network.

Overall, among our socioeconomic measures, employment status was most strongly associated with multimorbidity, while associations were less consistent for poverty and educational attainment. In part, the relatively small health inequalities in old age observed in this study may be due to the fact that some SEP measures may not sufficiently sense such inequalities among the Korean elderly. Household income measure may not accurately indicate the material status at old age for two primary reasons. First, the assumption of equivalent household income that the total income of all family members is evenly shared by each member is rather crude when applied to the elderly. Second, diverse income sources among older people are subjected to missing data on some details, which hinders the collection of accurate information [48]. Similarly, the use of education as an SEP indicator in old age also has some disadvantages. Elderly people in Korea mostly attained a lower-than elementary level of education. Thus, educational attainment may be insufficient to differentiate socioeconomic variation in this population. Additionally, education attained at early adulthood may be less influential to health at old age [49]. The use of employment status measure may have advantages over other SEP measures. First, unlike household income, employment status is measured at the individual level and thus is less prone to measurement error. Second, employment status is to assess operation of current status, whereas educational attainment represents a long-term influence from early life, the impact of which may dilute over time. This study supports the notion that examining multiple SEP measures in a comparable manner is necessary to demonstrate socioeconomic variation in multimorbidity and also shows that no single measure can provide a comprehensive picture in linking SEP to multimorbidity.

Among modifiable risk factors, associations were observed for cigarette smoking and obesity, particularly with higher number of chronic diseases across age groups (from the 50s to 70s). Associations for alcohol drinking and physical activity were less consistent (data not presented). This suggests that the associations between health behaviours and SEP are independent of each other. A few previous studies have considered the role of health behaviours in relation to health inequalities; individuals from lower SEP are more likely to have harmful health behaviours [50, 51]. Further studies are required to examine the mediation or independent role of health behaviours in the context of multimorbidity.

To conclude, the present study expands the scope of health inequalities research area and suggests that socioeconomic inequalities in multimorbidity vary according to sub-categories of multimorbidity. For example, the disease pairs that co-occurring with cancer demonstrated relatively smaller socioeconomic gradients. Furthermore, our study partly supports larger health inequalities at old age in circumstances, where socioeconomic disadvantages continue or even increase in later life as in Korea. Employing proximal SEP measure such as employment status in relation to multimorbidity is expected to show a potentially larger variation in health among the elderly.

## Supporting information

S1 Table Prevalence of 66 chronic diseases and 7 diseases and disease groups. (DOCX)

S2 Table Prevalence of co-occurring chronic diseases. (DOCX)

## Abbreviations

CVD: cardiovascular disease
SEP: socioeconomic position
CCC: chronic condition classification
OECD: Organization for Economic Cooperation and Development
