# Supplementary material for: Socioeconomic inequalities in health in the context of multimorbidity: A Korean panel study
Source: PLoS One. 2017 Mar 15;12(3):e0173770. doi: 10.1371/journal.pone.0173770 (PMC5351993; doi:10.1371/journal.pone.0173770)
Supplement: S1 Table — (DOCX) [file pone.0173770.s001.docx]

S1 Table. Prevalence of 66 chronic diseases and 7 diseases and disease groups

| No | Chronic diseases | N | % | Disease groups | N | % |
| --- | --- | --- | --- | --- | --- | --- |
| 1 | Cancer | 689 | 3.46 | Cancer | 689 | 3.5 |
| 2 | Schizophrenia | 45 | 0.2 | Mental disorder | 424 | 2.1 |
| 3 | Paranoid states | 3 | 0.02 |  |  |  |
| 4 | Obsessive-compulsive disorder | 2 | 0.01 |  |  |  |
| 5 | Panic disorder | 21 | 0.1 |  |  |  |
| 6 | Depression | 4 | 0.02 |  |  |  |
| 7 | Bipolar disorder | 14 | 0.1 |  |  |  |
| 8 | Anxiety disorder | 335 | 1.7 |  |  |  |
| 9 | Chronic obstructive pulmonary disease | 38 | 0.2 | Respiratory diseases | 560 | 2.8 |
| 10 | Chronic bronchitis | 111 | 0.6 |  |  |  |
| 11 | Emphysema | 15 | 0.1 |  |  |  |
| 12 | Bronchiectasis | 17 | 0.1 |  |  |  |
| 13 | Pneumoconiosis & asbestosis | 16 | 0.1 |  |  |  |
| 14 | Asthma | 363 | 1.8 |  |  |  |
| 15 | Acute myocardial infarct | 155 | 0.8 | Cardiovascular diseases | 1,334 | 6.7 |
| 16 | Angina Pectoris & other ischemic heart disease | 423 | 2.1 |  |  |  |
| 17 | Arrythmia | 116 | 0.6 |  |  |  |
| 18 | Heart failure | 38 | 0.2 |  |  |  |
| 19 | Stroke | 215 | 1.1 |  |  |  |
| 20 | Cerebral infarct | 307 | 1.5 |  |  |  |
| 21 | Other cerebrovascular disease | 80 | 0.4 |  |  |  |
| 22 | Diabetes | 1,852 | 9.3 | Diabetes | 1,852 | 9.3 |
| 23 | Hypertension | 4,887 | 24.5 | Hypertension | 4,887 | 24.5 |
| 24 | Alcohol dependence syndrome | 8 | 0.04 | Others | 12,830 | 64.3 |
| 25 | Drug dependence | 54 | 0.3 |  |  |  |
| 26 | Hyperkinetic syndrome | 2 | 0.01 |  |  |  |
| 27 | Sleep disorder | 353 | 1.8 |  |  |  |
| 28 | Parkinson’s disease | 62 | 0.3 |  |  |  |
| 29 | Multiple sclerosis | 1 | 0.01 |  |  |  |
| 30 | Paralytic syndrome | 14 | 0.1 |  |  |  |
| 31 | Epilepsy | 61 | 0.3 |  |  |  |
| 32 | Migraine | 137 | 0.7 |  |  |  |
| 33 | Glaucoma | 177 | 0.9 |  |  |  |
| 34 | Keratitis | 14 | 0.1 |  |  |  |
| 35 | Cataract | 1,128 | 5.7 |  |  |  |
| 36 | Hearing loss | 55 | 0.3 |  |  |  |
| 37 | Rheumatic fever | 1 | 0.01 |  |  |  |
| 38 | Atherosclerosis | 34 | 0.2 |  |  |  |
| 39 | Peripheral vascular disease | 16 | 0.1 |  |  |  |
| 40 | Chronic sinusitis | 135 | 0.7 |  |  |  |
| 41 | Chronic tonsillitis | 16 | 0.1 |  |  |  |
| 42 | Allergic rhinitis | 911 | 4.6 |  |  |  |
| 43 | Crohn’s disease & Ulcerative colitis | 12 | 0.1 |  |  |  |
| 44 | Diverticular disease | 4 | 0.02 |  |  |  |
| 45 | Fatty liver | 167 | 0.8 |  |  |  |
| 46 | Cirrhosis | 56 | 0.3 |  |  |  |
| 47 | Nephritis | 41 | 0.2 |  |  |  |
| 48 | Renal failure | 103 | 0.5 |  |  |  |
| 49 | Hyperplasia of prostate | 531 | 2.7 |  |  |  |
| 50 | Disorders of penis | 2 | 0.01 |  |  |  |
| 51 | Disorders of menstruation | 14 | 0.1 |  |  |  |
| 52 | Menopausal disorders | 289 | 1.5 |  |  |  |
| 53 | Atopic dermatitis | 268 | 1.3 |  |  |  |
| 54 | Connective tissue disease | 9 | 0.1 |  |  |  |
| 55 | Rheumatic arthritis | 240 | 1.2 |  |  |  |
| 56 | Degenerative arthritis | 2,682 | 13.5 |  |  |  |
| 57 | Inflammatory spondylopathies | 8 | 0.04 |  |  |  |
| 58 | Spondylosis | 498 | 2.5 |  |  |  |
| 59 | Intervertebral disc disorder | 1,169 | 5.9 |  |  |  |
| 60 | Osteoporosis | 1,256 | 6.3 |  |  |  |
| 61 | Congenital anomaly | 5 | 0.03 |  |  |  |
| 62 | Hyperlipidemia | 1,704 | 8.5 |  |  |  |
| 63 | Gout | 171 | 0.9 |  |  |  |
| 64 | Dementia | 84 | 0.4 |  |  |  |
| 65 | Acquired hypothyroidism | 193 | 1.0 |  |  |  |
| 66 | Thyrotoxicosis | 145 | 0.7 |  |  |  |
