# Supplementary material for: Socioeconomic inequalities in health in the context of multimorbidity: A Korean panel study
Source: PLoS One. 2017 Mar 15;12(3):e0173770. doi: 10.1371/journal.pone.0173770 (PMC5351993; doi:10.1371/journal.pone.0173770)
Supplement: S2 Table — (DOCX) [file pone.0173770.s002.docx]

S2 Table. Prevalence of co-occurring chronic diseases

|  | Cancer | |  | Mental disorder | |  | Respiratory disease | |  | Cardiovascular diseases | |  | Diabetes | |  | Hypertension | |
| --- | --- | --- | --- | --- | --- | --- | --- | --- | --- | --- | --- | --- | --- | --- | --- | --- | --- |
|  | N | % |  | N | % |  | N | % |  | N | % |  | N | % |  | N | % |
| No index disease | 19,253 | 96.5 |  | 19,493 | 97.8 |  | 19,406 | 97.3 |  | 18,695 | 93.8 |  | 18,090 | 90.7 |  | 15,655 | 75.5 |
| Single disease | 236 | 1.2 |  | 119 | 0.6 |  | 89 | 0.5 |  | 108 | 0.5 |  | 221 | 1.1 |  | 1,067 | 5.4 |
| Cancer | - | |  | 20 | 0.1 |  | 27 | 0.1 |  | 65 | 0.3 |  | 87 | 0.4 |  | 218 | 1.1 |
| Mental disorder | 20 | 0.1 |  | - | |  | 18 | 0.1 |  | 73 | 0.4 |  | 61 | 0.3 |  | 163 | 0.8 |
| Respiratory disease | 22 | 0.1 |  | 18 | 0.1 |  | - | |  | 72 | 0.4 |  | 67 | 0.3 |  | 203 | 1.0 |
| Cardiovascular diseases | 65 | 0.3 |  | 73 | 0.4 |  | 72 | 0.4 |  | - | |  | 324 | 1.6 |  | 754 | 3.8 |
| Diabetes | 71 | 0.4 |  | 35 | 0.2 |  | 67 | 0.3 |  | 306 | 1.5 |  | - | |  | 766 | 3.8 |
| Hypertension | 123 | 0.6 |  | 79 | 0.4 |  | 145 | 0.7 |  | 456 | 2.3 |  | 766 | 3.8 |  |  | |
| Others | 152 | 0.8 |  | 105 | 0.5 |  | 118 | 0.6 |  | 167 | 0.8 |  | 326 | 1.6 |  | 1,716 | 6.6 |
